# Anatomical Examination, Description, and Arrangement of Double Monsters

**Published:** 1841-10

**Authors:** 


### ART. V.

*Ontleedkundig Onderzoek, Beschrijving en Rangschikking der Dubbelde Missgeboorten*, door W. VROLIK, Med. Doct. en Prof.—*Amsterdam*, 1840. 4to, pp. 232, met 9 Platen.

*Anatomical Examination, Description, and Arrangement of Double Monsters.* By W. VROLIK, M.D. and Professor.—*Amsterdam*, 1840. With 9 Plates.

WE shall best express for ourselves and ensure from others the praise which this admirable monograph deserves, by giving an abstract of its contents, and by removing it from the obscurity in which its language hides it. The only preface we need make is, that whatever form of monstrosity we refer to is illustrated by one or more well-authenticated and clearly described examples of it, which have either occurred in the author's own researches or been taken from the works of others; so that an abstract can, in reality, be little more than a syllabus of the facts which are distributed in the richest profusion throughout the work.

Excluding from his present enquiries the greater number of the examples of the *fœtus in fœtu*, because in them the one body rather resides within the other as a foreign substance than forms a part of one common organic system, Dr. Vrolik regards as double monsters all those in which either a single part, or a whole system of organs is multiplied, more or less of the constituents of two bodies being united in one. He arranges all the members of this class in several principal divisions, the distinctions of which are based at once upon the prominent external and the most important internal characters; and within each class he again orderly arranges a number of subordinate forms determined chiefly by the extent and the complexity of the monstrosity in each.

I. The first division is that of *Heteradelphia ;* this name being adopted from Geoffroy St. Hilaire to indicate those double bodies of which the components are very unequal, and of which one part may be regarded as the stem or trunk to which another organized part, or even a whole body, less developed than itself, is affixed like a parasite.

Both the nature and the situation of this parasite are various. Most often indeed it is fixed to the epigastric region ; but it is also not unfrequently seated on the head, the pelvis, and many other parts of the body. On whatever part it is fixed, it is held there by no mere external adhesion, but by an intimate connexion of parts ; by blood-vessels and a loop of intestine, or some such important organic bond of union, as to constitute one being with the twin stem to which it is inseparably connected.

Examples of this kind of monstrosity have been published in considerable numbers. Arranged according to the nature and the seat of attachment of the parasitic portion, Dr. Vrolik refers to and describes cases in which a second head was fixed to the epigastrium, to the back, to the skull, the vertex, and the lower jaw ; others, and these more numerous, in which a pelvis and lower extremities seemed to grow from the walls of the abdomen, or from the other pelvis; others in which superfluous upper or lower (anterior or posterior) extremities were connected with the front wall of the abdomen, with the back, the side of the chest, the head, or the other limbs; others, again, in which the appended parasite was a headless monster with four limbs; and lastly, in a fourth class, the cases in which it consisted of a complete individual with a head and four limbs attached to the epigastric or the pubic region of its more developed fellow.

The common character by which this whole division is distinguished from other double monsters is the comparatively smaller size, and, in general, the defective development of the part which is termed the parasite. Imagine this difference removed by the fuller development of the parasite, by its obtaining all its own organic apparatus, and by its growing *pari passu* with the other, and an exact idea of complete duplicity* will be formed. It will be observed also, (and this is a point especially to be borne in mind both in this and in all the other divisions,) that in the several members of this class there is a regularly graduate series from those in which the superfluous part is only an ill-developed limb, to those in which the parasite differs from the stem in nothing but its inferior size, and its dependence for nutrition. The cases of the last kind however are rare; Dr. Vrolik has been able to find but three ; much more commonly the parasite, even when it possesses its full numerical

* The term may appear strange ; but our language has no other adapted to the purpose, and its use in this sense is warranted by the similar employment of *simplicity* in descriptions of both moral and physical characters.

complement of parts, bears many signs of defective development; it is hare-lipped, or a cyclops, or has atresia ani, or some other malformation by arrest of development. And this again is a fact of much importance to the author's theory of duplicity, which depends in great measure on the proof that in these heteradelphs there are always the rudiments of two bodies, though one or both may be defective.

The beings thus formed have rarely lived many years after birth, and the histories of the few that have survived are for the most part well known in the records of medicine. Perhaps the most remarkable is that of the Chinese A-Ke, of whom and his parasite, the little models are to be found in most of our anatomical museums, and who is described by Chapman in the Philadelphia Journal for November, 1821. The parasite's life is in general only vegetative. In one of the three cases, indeed, in which it possessed all the constituent parts of a body, it moved its limbs and appeared to have its own sensations, though it neither ate nor discharged fæces or urine; but in the others less perfectly formed, even these signs of individual life were absent, and in only one, that of the Chinese A-Ke, had the man who bore the parasite any voluntary power over its limbs. The nutrition of the parasite appears to depend entirely on the stem-body to which it is fixed, and through which it both receives its nutritive materials, and discharges its excretions. The one increases and decreases in size with the other; and, of course, the parasite dies with the stem. The influence which, in its turn, it exercises on its supporter is not always important. In the heteradelphi that die early, death commonly ensues from the ill formation of the main body; if they survive, the parasite seems to do harm, only as an ordinary tumour would, by its weight and by abstracting a certain amount of nourishment, so that those who, thus burdened, have grown up to childhood or manhood have usually been thin and delicate like men subject to some unnatural waste. At the same time, the author observes, it will always be far better to tolerate this evil than to risk an operation of removal, when the results of all the examinations yet made prove that the parasite is deeply and by important organs connected with the stem.

Of the general correctness of the author's opinion on this subject there can be no doubt; but exceptions must be admitted. In a very interesting paper on Congenital Tumours of the Pelvis, lately read by Mr. Stanley at the Medical and Chirurgical Society, a case was related in which Mr. Blizard removed with complete success from the sacrum of a child a large mass in which was included an isolated portion of intestine. There was no doubt therefore of this being a *parasite*, although it presented no external resemblance to the form of a second child. The cases described in Mr. Stanley's paper afford examples of duplicity with which the author seems unacquainted. In all of them perfect children were born with large masses composed of tough fibrous cysts containing fluid, and of firm masses like the ordinary fibrous tumours of the uterus, attached to the lower part of the trunk. In two of them there were isolated portions of intestine filled with a substance like meconium.

II. It has been already said that some of the rarer kinds of the heteradelphia approximate closely to the completely double monsters. In all the cases that stand nearest to the transition, the parasite has been found adherent to the epigastric region, and the kind of duplicity which is most closely related to them is therefore that in which the two bodies adhere by their anterior surfaces,—the kind which Dr. Vrolik has termed *Verdubbeling van voren*, or, as we may be allowed freely to render it, *Anterior Duplicity*.

The most complete examples of duplicity yet known are found in this class, whose distinctive characters are, that two bodies in a state of nearly equal development are placed exactly opposite to one another, with their sterna connected together, and with their abdominal cavities either partially or completely confounded. Here, however, as in all the other classes, examples are found of gradations towards a state of singleness. Such are those in which the upper parts of the body being completely double, the lower are united so that there are but three, or two, lower (or posterior*) limbs. And, in like manner, although in many cases the two bodies are alike in size and other characters, yet there are many more in which one has so far surpassed the other both in size and measure of development, as completely to fill up the series between this class and the decided heteradelphs. While in the former class we find the closest approximations to duplicity, in this, and even among the most perfect of the double monsters, there are peculiarities which constantly recall to mind the parasitic attachment of the others.

With this nearly perfect external duplicity there sometimes corresponds an equal duplicity of internal organs. The bond of union, as far as the skeleton is concerned, is commonly a tough fibrous connexion between the lower extremities of the sterna and the ensiform cartilages which are set directly opposite to one another. The rest of the sterna and the ribs are usually distinct, and the thoracic cavities are thereby separated. In this case there are commonly two separate and perfect hearts, but in the cases in which the sterna are less completely separated, or (as happened in one case) entirely absent, only a single heart, or one partially doubled, with, for instance, two ventricles and four auricles, or otherwise malformed, is found. But it is particularly remarkable that in these, as in all other kinds of double monsters, there is no constant relation whatever between the respective states of the external and the internal organs, for the condition of the two digestive canals, even in those who are externally almost exactly alike, is subject to still greater varieties than the condition of the heart. The abdominal organs are always in some degree connected; the two livers are usually continuous; a spleen, pancreas, and stomach are commonly found in each body, and each stomach has its own duodenum, which after some length being continued into jejunum, unites with the other to form a single tract of small intestine, which again divides into two canals which lead respectively to the large intestine of each body. The lungs, and urinary and genital systems are always double.

The most remarkable example of this class was the well-known Siamese Twins; when exhibited they were not exactly opposite to each other, but stood side by side, or rather, obliquely one by the other; but this position, there can be little doubt, was acquired by the attempts which they

* The examples cited in this and all other parts of the work are taken indifferently from human bodies and those of lower animals; for brevity's sake, however, we shall use the terms *upper* and *lower*, when applied to the limbs, as synonymous with *anterior* and *posterior*.

had instinctively made to separate from each other in walking, or in lying and sitting down, and by the extension they had thus effected in their bond of union, which was considerably more slender than in any other yet described. From the analogy of several similar cases, Dr. Vrolik thinks it most probable that these youths were connected by the ends of their sterna and by some of their abdominal organs; and in discussing the question often agitated in this country while they were exhibited, whether any separation of them should be attempted, he introduces the figure and description of a double fœtus, which very closely resembled them in all the external circumstances of its duplicity. In this the two diaphragms met in the bond of union, and were connected by a kind of third central tendon at the points of the united ensiform cartilages. The peritonea were separate; but from each of them there was sent down near the middle of the bond of union a prolongation forming a kind of coronary ligament to a considerable portion of liver which passed across the bond and connected the main masses of the livers of each fœtus. All the other organs were completely separate.

As far as the Siamese youths were concerned, the question of operation was set at rest by the observation of Mr. Mayo, communicated at the Conversazione of the College of Physicians, March 8, 1831,* that when either of the youths coughed the bond of union swelled up in its whole length, proving that they had but one peritoneal cavity of which a transverse prolongation passed bridge like through the connecting medium. And in every case the probability of having to cut through a piece of liver or a peritoneal canal, must render an operation unwarrantable, unless indeed after the death of one of the bodies during the health of the other. The case reported by König† has scarcely authority enough to support a contrary opinion.

III. The varieties of form in anteriorly-doubled monsters are closely limited by the partial union of the sterna and the general separation of the thoracic cavities, and (through their medium) of the whole upper part of the body. In the next class, the examples of which are distinguished by the name of *lateral duplicity*, there is no such limit; and between the highest degree of duplicity found in it and the lowest, or that in which the duplicity is most nearly reduced to singleness, there is a far more numerous series of intermediate forms than in any other of the divisions.

"In lateral duplicity the two bodies are not set opposite to one another, but are turned sideways from one another. They have a common thoracic cavity, for the formation of which (at least in the highest degree of duplicity) the right ribs of one body and the left of the other proceed towards the anterior and posterior aspects, and are there connected with an anterior and posterior sternum. The best idea of the construction of this osseous fabric may be formed by supposing the two complete chests of two bodies to be set one against the other, and that then the anterior extremities of the right ribs of the right body, and those of the left ribs of the left body unite with one sternum and pull it forwards, while, in the same manner, the left ribs of the right body and the right of the left unite on the posterior aspect with the other sternum and carry it backwards. The consequence is that the two vertebral columns are turned away from one

* See the Medical Gazette of that month.
† Ephem. Nat. Curios. Dec. 2, ann. viii.

another, and that all the parts above and below the thorax are double. (p. 75.) .......... By the formation of this common thorax the lateral is completely distinguished from the anterior duplicity in which the thoraces are (*commonly*) connected only by the points of the sterna, and, as to their cavities, are separate." (p. 173.)

And with these differences of external construction, others not less important of internal arrangement coincide, which fully justify the separation of the two forms, however similar the external appearances of many of the examples of either may be.

The varieties of lateral duplicity, which are so numerous that their brief description occupies nearly half the book, may be divided into two principal sets. The first begins with the complete duplicity of the whole body and ends with its perfect simplicity; in the second the duplicity of the body remains, but the head gradually becomes single. The forms included herein may be briefly summed up as follows:

1. Complete duplicity, with all the external parts, and sometimes the abdominal and pelvic organs double, and with one common double-sized thoracic cavity formed in the manner just described, and containing four lungs and (in all four cases) only one heart. The examples of this form are very numerous, and are to be met with in all our large museums.

2. In the examples of this second group, which exhibits the first step towards simplicity, one of the sterna may be traced in a succession of specimens becoming gradually narrower, and permitting a closer approximation of the two corresponding upper extremities, till, in some examples, they are completely united, and there are found only three limbs above with three or four below. The two scapulæ on one aspect of the bodies, for example, are merged into one; or there are two scapulæ on each aspect, but one pair of them have only one humerus between them, and this splits below to articulate with two lower arms; or there is but one lower arm, and this bears supernumerary fingers. In some way, the varieties of which are very numerous,' there is a tendency towards union of two of the upper extremities.

3. In the third group we have a repetition of the same series of changes in the lower limbs as in the second was traced in the upper; here, as there, presenting numerous varieties, in the last and lowest of which only three lower limbs, and the third of these ill formed, are found.

4. The third limb has now gradually disappeared, and with a complete duplicity above the pelvis, there are but two limbs below it, and these well formed. In this class are placed with many others the Ritta-Christina monster described by M. Serres, which lived to the eighth year, and the still more remarkable example mentioned by Buchanan of a two-headed man twenty-eight years old, who lived in the reign of James the Third of Scotland.

5. The union proceeding, and this simplicity of the lower part of the body being retained, examples come next in which the upper parts also are united; the two superfluous upper limbs being united into one, presenting a single upper arm with a double fore-arm and hand, or a single upper arm, fore-arm, and hand with ten figures, or only a malformed limb, or a mere projection occupying the place of the superfluous limbs.

6. Even this last indication of duplicity of the upper parts ceases, a scapula only remains, or this also is absent; and next, one of the sterna having disappeared and the vertebral columns having been connected on the corresponding side by their respective ribs united into single arches, these now become gradually shorter, and the columns approach each other more and more nearly till they are connected by only a cartilaginous substance in the place of ribs, or are at some part confounded.

7. In this next group both the upper and lower parts of the body are single; the vertebral column is single all below the cervical region, or exhibits only a trace of duplicity (to which something similar is often presented by the sternum), but at the cervical region becomes double, and on each portion bears a head. Of this also there are many examples published, and from them there is a gradual transition through the eighth group in which the portion of the cervical part of the column that is double becomes less and less, to the ninth in which the two heads are seated on an apparently single neck in which all the cervical vertebræ are simple, or only bear traces of duplicity, except the first two or the first alone.

10. Hitherto the duplicity of the head was perfect; in this group the two heads also begin to coalesce, and in a considerable number of cases referred to, gradations are traced in which the adjacent ears are very closely approximated, and the heads are united behind; then, in which one ear only is placed between the adjacent surfaces of the two heads, and this gradually and at last totally disappearing. Next in order are the cases in which the adjacent ears being lost the two adjacent and middle eyes first become very close and then are confounded in one cavity; then those in which there is such a union of the heads that the two upper jaws are articulated with one lower jaw; and lastly, those in which the head is doubled only in individual parts, or in which there is one perfect head with some imperfect part or parts of another attached to it.

11. The eleventh group of this division includes the cases of lateral duplicity in which the body is single in the middle but doubled above and below (or, in brutes, anteriorly and posteriorly). In these, which are of rarer kinds, a single neck bears two more or less completely separated heads. The vertebral column is for a considerable length single, but at its lower part again divides and bears two sets of lower extremities.

12. In this the body is single above and doubled below. In the 13th there is tendency towards or even complete singleness of the head, but all parts of the trunk and all the limbs are doubled, an arrangement by which, as already stated, these form a series entirely distinct from the rest. In some of these cases the two heads are found coalesced below; in others, to which the name of janiceps has been given, one face is directed backwards and the other forwards, the remainders of the two heads being merged into one; in others, one face is well, the other very deficiently developed; in others there are only the indistinct traces of a second head presented in the existence of one or two ears on the posterior aspect of the more perfect one; in others this trace of duplicity is still less evident; and lastly, in the remainder of the group it is entirely lost, and one head only, and that well formed, is found upon the double body.

IV. The fourth great division which Dr. Vrolik has made includes the cases in which there are two complete bodies with the lower portions of their respective trunks united, so that there is a head with upper extremities both above and below (the bodies being placed in the same straight line), and on either side of the part at which they meet two lower limbs. One may best conceive this arrangement by supposing two children stuck together by their buttocks, and so fixed with wide-spreading lower limbs. A common body is thus formed with a head at each end, with two upper limbs both above and below, and with two lower limbs, one belonging to each fœtus on the right, and two on the left of the united portions. A few cases only of this remarkable monstrosity are recorded; and in these the duplicity was not always complete, but exhibited in some the same tendency towards simplicity as was noticed in the others. Thus in some there are but three lower extremities; in others there are but two, or two with a third ill-developed on the other side; and again in other groups there are those which have a perfect head at one end of the trunk but an imperfect one or none at all at the other.

These monsters have been known to live a considerable time; their capacity for life being probably owing to the separation of the hearts and the absence of malformation in the more important organs of the body. The umbilical cord is single and never has a double set of vessels; an apparent proof (confirmed by the similar examples in other classes), that the one body is not formed of the materials of two, a conclusion which is supported by the coincident singleness of the anus and urinary bladder, and the union of the intestinal canals.

V. The fifth chief form is the *posterior duplicity* in which two bodies are united by their backs or a part of them. The union may be at the pelvis (which is most common, and had occurred in the well-known Hungarian sisters, who lived to their twenty-second year), or at the back of the vertebral column.

VI. The sixth is the *superior duplicity*, in which the two children are connected by their skulls, the bones of which are united so as to form a single cavity. In these also the place of union varies greatly. The frontal bone of one coalesces with the malar or the occipital bone of the other, or the foreheads are attached to one another, or the side of one head to the front of the other. But all these are very rare, and of each kind only one or two examples can be found on record.

VII-VIII. With the last division is concluded the description of the true double monsters. A seventh division, however, is added to take in the *triple-bodied* monsters, of which one instance only is known to have occurred in the human subject;* and an eighth to include the numerous cases of duplicity of individual parts of the body, the rest being single. Instances of this are found in parts about the head, chest, abdomen, and limbs; such as, for examples, two mouths, supernumerary teeth and horns, two œsophagi or duodena, double hearts, or supernumerary cavities in one otherwise well formed, a double penis and urethra, a double clitoris, supernumerary breasts, kidneys, vertebræ, ribs, fingers, toes, or whole limbs.

Such is a brief account of the facts of Dr. Vrolik's work; we now proceed to point out some of the generalizations of which they evidently

* Atti dell' Academia de Cattania, t. viii. 1834.

admit, and the opinions respecting the origin and production of double monsters which they seem to support.

The double monsters form collectively one class of organic beings, which, however different in their several degrees of malformation, may be arranged in one continued series. As the lowest degree of duplicity may be mentioned that of a single part of the body, for example, a double or supernumerary finger; as the highest, a complete double monster with two heads, four upper and four lower limbs, and two trunks, such as the Siamese twins formed. And between these two extremes there are different forms of duplicity which gradually run one into the other.

There is no positive or constant relation between the external and the internal organs as to their degrees or modes of duplicity. In the completest duplicity of the exterior, for example, the heart is often single or even shows signs of having been arrested in its development; and on the other hand in the more nearly simple forms the heart is usually either partially or completely double. The histories of the cases of anterior and lateral duplicity furnish abundant proofs of this. Nor is there any closer relation between the condition of any other internal organ and that of the exterior than there is between the heart and it; for in nearly complete external duplicity any of the internal organs may be single. When there are two trunks, indeed, the urinary and genital organs are commonly double; but as for the stomach, the liver, and the lungs, the correspondence between them and external duplicity follows no other rule than that where there are two necks there are two tracheæ and two œsophagi, and through their medium the lungs and stomach are also doubled. When, in like manner, the stomach is double, each has its spleen and pancreas; but the state of the liver is very variable; sometimes there are two, sometimes but one with a single or a double gall-bladder; and these differences often occur in the same form of external duplicity.

Parts placed on the surface of the body are more liable to multiplication than the internal organs, and duplicity of a single part is therefore much less rare than the formation of a complete double body. The upper half of the body is more frequently doubled than the lower, probably in consequence of its earlier development, and the admitted preponderance of the upper parts of the body. The union of the two bodies takes place only between similar parts. The more each of the bodies is developed, the less is the bond of union between them, as the examples of the Siamese twins and the Hungarian sisters sufficiently prove. And with this law is connected another, namely, that the probability of growing up is greater in the same proportion as the bond of union is smaller, and the coincident confusion of internal organs less, as the two same double monsters, and others besides them prove. So also, the further the several organs are from the situation at which the bodies are united, the more perfect they are,—one body is almost always less developed than the other; in the heteradelphs this is always the case, and in others the difference between the two bodies, though less evident, is scarcely less constant than in them.

There are not commonly any signs of a double monster having been at first two individuals. For except in the cases of posterior and superior duplicity, and some singular examples of attachment of the umbilical cord of one fœtus to the head or body of the other, there is never more than one placenta and one cord, and the latter usually contains only a single set of vessels, which divide when they reach the abdomen. And even in the posterior and superior varieties of duplicity it is not yet certain that there are two placentæ; in some cases the placenta was positively single, and in the remainder it has very rarely been examined.

And after all these facts, "what explanation," says the author, "is to be given of the origin of double monsters? The answer may be brief,—none," (p. 219.) It is not difficult to find in his volume of facts, enough to cast doubt on any hypothesis; yet the subject acquires so much more interest by being treated as capable of explanation than it has in its mere detail of facts, that we must undertake the consideration of the probable origin of double monsters, even at the risk of "wading in the region of guesses," from which the author, though he adopts the same views as we do, almost holds himself aloof.

The choice seems to lie between the three hypotheses, to which, variously expressed, nearly all ever suggested may be reduced, namely, 1st, that of originally double ova; 2d, that of an excess or wrong distribution of formative power in a single ovum; and 3d, that of the adhesion and fusion of two single ova. On the comparative merits of the first and third, as parts of the general doctrine of monsters, one of the most interesting physiological discussions extant is recorded in the Mémoires de l'Acad. des Sciences de Paris, between 1724 and 1743. The chief disputants were Lemery and Winslow; the contest lasted nineteen years; it engaged the attention of all anatomists, and called forth writings by Haller and a crowd of authors of less note, and was only terminated by the death of Lemery. Every argument that could be founded on the knowledge of those days was brought forward, and the subject was, for the time, utterly exhausted; but the facts accumulated in later years have furnished such volumes of additional evidence, that the same question between original and acquired monstrosity, as far as it relates to double monsters, may even now claim to be discussed.

The first hypothesis, which supposes an original formal fault in the ovum, is inconsistent with the fact, that in its earliest periods the ovum has no relation as to its form with the being subsequently produced, whether naturally or unnaturally. It contains in miniature no one of the organs of the fœtus: it is a mere cell, including two or more kinds of other smaller cells which in part form, but in greater part elaborate, the perfect organs. But none of these cells have any formal relation to the organs, *they* are contained only *potentially* in the ovum. When therefore we know that in an ovum there is neither head nor heart, trunk nor limb materially present in miniature form, it is not imaginable that there should be such a partial substantial doubleness of an ovum as should produce two heads or two hearts, or any other organ twice instead of once. When no organs are formally present single, there, *à fortiori*, there can be none double. A material duplicity of an ovum could only consist in an additional number of its homogeneous cells; but the result of such a condition would probably be, not a numerical increase, but only an unnatural size of the organs.

The second hypothesis agrees with the first, in that it admits the probability of an original fault in an impregnated ovum being the common precedent of duplicity, but differs from it chiefly in supposing this fault to be one, not of form but of power; and in supposing further that the fault may be either original and inherent, or the result of some extraneous influence.

There is one fact which must be mentioned at once, because it establishes fully that monsters of all kinds are at least sometimes the products of ova originally, or by impregnation rendered anormal, and thus decides the claim of this second hypothesis to a fair consideration to be greater than it has been commonly regarded. This fact is, that monstrosities are often hereditary, which it is obvious they could not be if their production always depended on some occurrence subsequent to impregnation. The occurrence of the same or similar defects in several members of one family, for example, must be the consequence of a fault in the parents conveyed to each several embryo. If such defects are similar (as they often are) to those existing in the mother, they must be ascribed to faults inherent in the ova; if they resemble deformities in the father, they must be regarded as due to faults in the semen; or if both parents be well formed, still the coincidence of malformation in several of their offspring cannot be regarded as accident, but must be ascribed to some fault of relation between the materials for reproduction contributed by each.

Having thus established that original fault in an impregnated ovum may be the foundation of monstrous productions, we must next point to the important fact determined by Dr. Vrolik, that double monsters form one series, among whose several members the degrees and modes of deviation from simplicity gradually increase, and pass without one abrupt step from the addition of a single ill-developed limb to the nearly complete formation of two perfect beings.* Now, if this be true, no hypothesis can be acceptable if it do not plausibly explain the origin of the whole series of double monsters, or if, though it may suffice to explain the facts in one part of the series, those in another part are opposed to it. And here is a fair objection against the hypothesis of two originally perfect germs. Grant that by it we might explain the formation of the Siamese twins, for example, by supposing that the opposite parts of the vitelline membranes, and then of the ventral arches of two normal ova became adherent and were fused, or that a similar contact of ova with their dorsal or ventral arches still open for a greater or less extent might explain (if we can imagine its occurrence otherwise than by accident) the formation of several of the more perfect instances of duplicity; still, if the same hypothesis is altogether opposed by the simpler forms of duplicity, it is surely not tenable. It may explain the union of two nearly complete digestive canals, but it cannot account for the existence on a child's sacrum of a shapeless mass, containing an isolated portion of intestine, as in Mr. Stanley's cases. And still less can it explain the

* This fact is one of many proofs, that error lies at the very foundation of M. Isidore St. Hilaire's system of teratology, which is based on the importance of the several deviations to the life or comfort of the individual. On this rule he separates widely the lower from the higher degrees of duplicity. But relative importance depends on the degree, not on the kind, of these monstrosities, and therefore cannot form the basis of a sound arrangement.

existence of a superfluous limb; for the limbs are mere off-shoots, and are produced at so late a period, that if we could imagine two embryos to come in contact by their shoulders or pelvis, and a fusion of those parts to take place, we should still have to explain how one of them leaving only an arm or a leg behind him, could, for the rest of his substance, head, trunk, and all, wholly disappear.

This difficulty lies in the way of the explanation by fusion of all the simpler forms of double monsters. For although recent observations have removed the main objections that Lemery had to contend with, by proving the possibility and even the facility with which two embryonic organs when brought together may be fused into one which will exhibit parts of both, yet we know of nothing that renders it at all probable that any extent of fusion should altogether swallow up a considerable portion of any embryo already formed. On the contrary, in such fusion as is seen in single monsters, the Cyclopes for example, the parts fused are united, but in no degree destroyed, each seems to preserve nearly the condition which it had at the moment of contact.

But there is no such difficulty if the hypothesis of excess of power be adopted. All the organs exist in the impregnated ovum solely *in potentiâ*, and we can imagine no other explanation for the production of them in superfluity than an excess of that power.* Indeed this is commonly admitted for the examples of supernumerary members, and for the whole class of monsters by excess (as they are called), by those who for other cases of duplicity are fusionists.† We contend further, that different degrees or quantities of excess of power lie at the origin of all the cases, the degree of excess determining in each the degree of duplicity. If a certain excess of power be admitted capable of producing any one case of duplicity, other amounts of excess may be believed capable of producing all the other cases which differ from it only in degree; and Dr. Vrolik's work demonstrates that all double monsters may be referred to differences in degree of deviation from the normal simplicity.

But it may be said that something more than excess of power is needed to produce any given double monster; for although it might determine the production of something superfluous, it could not determine whether it should be an arm or a leg, or the whole upper or lower half of a body; nor could it alone render these monsters so regular as they are, but arms might grow from a pelvis, and legs from a head, and so on. We should answer that diversities of power in an ovum do not of necessity carry with them tendencies to deviate from the directions in which that power usually acts, although the heteradelphs show that they sometimes do. In normal development we are obliged to assume that the definite power in the ovum has to follow definite directions; but alterations in the amount of the former may surely exist without any change in the latter, nor can

* It would seem pedantic to use *potentia* instead of *power;* but in modern physiology the former usually implies not power only, but power with a tendency to the production of a definite effect; and in this view we shall use the term *power*, and the related adjective *potential*.

† M. Serres' early idea of the extra-development depending on the accidental (?) branching of an artery which then becomes the main artery, and in plain terms the producer of the new part is certainly incorrect. The blood-vessels are formed subsequently to the parts which they afterwards supply with the material necessary for their maintenance.

we tell why the normal power of an ovum always produces parts in their right places any more than why a power greater in degree does. Only on the whole, when the power in an ovum is absolutely greater than is natural, it is, *à priori*, much more probable that supernumerary parts will be produced in or near their right places, than that they will not.

In this last respect our hypothesis has the advantage of its rival. It was a main objection against the doctrine of Lemery that if two germs came in contact by accident (as he supposed) they could not exhibit any regularity in their mode of attachment, but faces would be forced into chests, abdomens into spines, and so on. The moderns who adopt the same hypothesis suppose that the ova come in contact, not by accident, but by an *attraction de soi pour soi*, of which the influence is that the two ova being by accident set face to face, or back to back, or in any way *similia similibus* will be drawn to each other, and will unite by similar parts. But, with all respect for the authority of M. Geoffroy St. Hilaire and his disciples, who regard this as "la règle suprême de tous les arrangemens et de toutes les modifications organiques chez les êtres composés,"* we confess that we can find no good evidence that such an *attraction* exists. We can see in it nothing more than a very happy expression of a fact which it in nowise explains. The extraordinary notion of MM. Delpech and Coste that such an attraction may be the result of electric currents is certainly no evidence of its existence, theirs being entirely imaginary. And the reasoning in its favour seems no better than the facts, for we can find nothing but this kind of circle; monsters adhere by similar parts; therefore there is an attraction de soi pour soi, there is then such an attraction, and therefore double monsters so adhere. We believe therefore that this attraction is hypothetical, and if it be so, surely the hypothesis which involves it and an accident as essential elements is less probable as well as less sufficient than that which we maintain. It is scarcely better than Lemery's of mere accident, for it requires not only the accident of a particular position of the ova, but that of their being of the same sex, for it is an almost invariable rule that both of the moieties of a double monster are either male or female, a fact which plainly agrees better with the notion of a single ovum possessing potentially certain sexual characters, than with that of two ova accidentally brought together.

We have assumed hitherto an absolute excess of power, but many double monsters exhibit excess in one part and defect in another, so that we must suppose in our hypothesis that in these cases the power, more or less excessive in quantity, is also wrongly distributed. Nor is this inconceivable, for since in the normal developmental power we must imagine at least two elements, *quantity* and *distribution*, and must acknowledge that for the attainment of a perfect result, the quantity must be distributed in definite proportions to each part; so it is not improbable that in certain circumstances of fault in the ovum, a normal or excessive power may be distributed disproportionately in the several parts.† But in the hypothesis of fusion the same facts are not clearly explicable. If

* Isidore St. Hilaire, Histoire des Anomalies, t. iii. p. 530.

† Sometimes both parts of a double monster, though well formed, are very small; this, however, is the result of defective *growth* which has no necessary relation to the development.

two embryos are fused by their ventral arches there is no evident reason why their dorsal arches should not be normally developed, for these are the materials for two perfect children, of which less than the quantity necessary for both are employed in the formation of their common inferior cavity. And in like manner in all similar cases, there is no reason why two healthy ova by contact should lose power. The only probable explanation, if the hypothesis of fusion be adopted, is to suppose that faulty ova are more likely to come in contact than healthy ones, but the maintainers of that theory would probably not cede so much to those who hold that ova are often originally faulty.

We have already mentioned the best proof that these original faults do sometimes exist, and it will be found that the objections commonly urged have but little or no force against their being the precedents of most or all cases of duplicity. For although it is fair to require that the same hypothesis should serve for the whole series of double monsters, because they differ only in degree, yet it is not reasonable to expect that both this series and those of monsters by defect of development and by malposition of parts should have a common mode of origin. The members of one series may most frequently be formed from ova, faulty in power, and those of others from ova originally healthy; and the arguments against the general notion of original faults may be valid in their relation to the one series, but utterly invalid when applied against the other. For example: the fact that accidents in the course of pregnancy, such as external injuries, mental impressions, and the like, sometimes give rise to the production of monsters, is of no weight against our hypothesis of the formation of double monsters, for such accidents are not (except by coincidence) followed by formation of double monsters. In the whole series of experiments by M. Geoffroy St. Hilaire, it is expressly stated that no instance occurred in which the disturbance of an egg during incubation resulted in the production of a double monster. Yet the influence of these accidents is the chief evidence against the hypothesis of original fault in germs, and to the cases in which their influence is exerted they are applicable enough. But for these double monsters which are not referrible to the influence of accidents, we can find no valid argument against the general supposition that they are derived from faulty ova; and, we may add, that even if an accident were occasionally proved to have influence in producing them it would not disprove our hypothesis. It is not essential to it that the germ should have a tendency to duplication, either only before or only directly after impregnation; the possibility is not denied that at any period of development an accident may give rise to a greater energy of the productive power; but it is rather in our favour that such accidents are more commonly, if not always, followed by defect. They prove the power to be a thing alterable and subject to changes dependent on external influences, and that which by one circumstance may be depressed, it is imaginable may, by another, be increased.

To sum up, therefore, our reasons for rejecting the hypothesis of fusion of ova, in favour of that of excess or irregular distribution of developmental power, for preferring (to use Dr. Vrolik's expression) to regard them as examples rather of singleness tending to duplicity, than of doubleness tending to simplicity, they are briefly these: that it is probable the whole class of monsters by excess (including those commonly so-called, and those usually regarded as double monsters) owe their origins to different degrees of one common fault; and consequently that the explanation of their origin ought to be the same for all; that no kind of fusion can account for the production of supernumerary individual organs, the rest of the body being single; but that it is not impossible that excess of power in one ovum, which all admit can alone explain the lower degrees of duplicity may, in proportionally higher degreee, produce the more completely double monsters, or even two such separate individuals as are sometimes found within a single amnion.

---

